# Bis(2,4-dioxo-5,5-diphenyl­imidazol­idin­ido-κ*N* ^3^)bis­(propane-1,3-diamine-κ^2^ *N*,*N*′)cobalt(II)

**DOI:** 10.1107/S1600536811050252

**Published:** 2011-11-30

**Authors:** Xilan Hu, Qing Jiang, Daqi Wang, Haifeng Liu

**Affiliations:** a Huaihai Institute of Technology, Jiangsu 222005, People’s Republic of China; b College of Chemistry and Chemical Engineering, Liaocheng University, Shandong 252059, People’s Republic of China

## Abstract

The complex mol­ecule of the title compound, [Co(C_15_H_11_N_2_O_2_)_2_(C_3_H_10_N_2_)_2_], has crystallographically imposed inversion symmetry. The Co^II^ atom displays a distorted octa­hedral coordination geometry. In the phenytoin anion, the two phenyl rings form dihedral angles of 62.26 (8) and 57.47 (9)° with the central imidazole ring. Intra­molecular N—H⋯O and C—H⋯O hydrogen bonds occur. In the crystal, N—H⋯O and C—H⋯O hydrogen bonds forming a three-dimensional network.

## Related literature

For applications of phenytoin, see: Milne *et al.* (1999 ▶); Akitsu *et al.* (1997 ▶); Akitsu & Einaga (2005 ▶). For related structures, see: Hu *et al.* (2006 ▶, 2007 ▶, 2009 ▶)
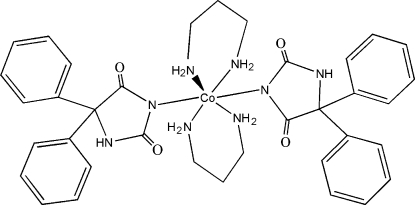


## Experimental

### 

#### Crystal data

[Co(C_15_H_11_N_2_O_2_)_2_(C_3_H_10_N_2_)_2_]
*M* *_r_* = 709.71
Monoclinic, 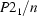

*a* = 10.0368 (12) Å
*b* = 8.7865 (9) Å
*c* = 20.684 (2) Å
β = 102.363 (2)°
*V* = 1781.8 (3) Å^3^
*Z* = 2
Mo *K*α radiation
μ = 0.53 mm^−1^
*T* = 298 K
0.50 × 0.46 × 0.45 mm

#### Data collection

Siemens SMART CCD area-detector diffractometer
Absorption correction: multi-scan (*SADABS*; Sheldrick, 1996 ▶) *T*_min_ = 0.777, *T*_max_ = 0.796
8632 measured reflections
3130 independent reflections
2536 reflections with *I* > 2σ(*I*)
*R*_int_ = 0.026

#### Refinement

*R*[*F* ^2^ > 2σ(*F* ^2^)] = 0.036
*wR*(*F* ^2^) = 0.097
*S* = 1.05
3130 reflections
224 parameters
H-atom parameters constrained
Δρ_max_ = 0.24 e Å^−3^
Δρ_min_ = −0.22 e Å^−3^

### 

Data collection: *SMART* (Siemens, 1996 ▶); cell refinement: *SAINT* (Siemens, 1996 ▶); data reduction: *SAINT*; program(s) used to solve structure: *SHELXS97* (Sheldrick, 2008 ▶); program(s) used to refine structure: *SHELXL97* (Sheldrick, 2008 ▶); molecular graphics: *SHELXTL* (Sheldrick, 2008 ▶); software used to prepare material for publication: *SHELXTL*.

## Supplementary Material

Crystal structure: contains datablock(s) I, global. DOI: 10.1107/S1600536811050252/rz2670sup1.cif

Supplementary material file. DOI: 10.1107/S1600536811050252/rz2670Isup2.mol

Supplementary material file. DOI: 10.1107/S1600536811050252/rz2670Isup4.mol

Supplementary material file. DOI: 10.1107/S1600536811050252/rz2670Isup5.cdx

Structure factors: contains datablock(s) I. DOI: 10.1107/S1600536811050252/rz2670Isup7.hkl

Additional supplementary materials:  crystallographic information; 3D view; checkCIF report

## Figures and Tables

**Table 1:** Selected bond lengths (Å)

Co1—N2	2.1531 (16)
Co1—N4	2.165 (2)
Co1—N3	2.180 (2)

**Table 2:** Hydrogen-bond geometry (Å, °)

*D*—H⋯*A*	*D*—H	H⋯*A*	*D*⋯*A*	*D*—H⋯*A*
N3—H3*A*⋯O1	0.90	2.35	3.061 (3)	136
N4—H4*B*⋯O2	0.90	2.38	3.095 (3)	137
C5—H5⋯O2	0.93	2.46	3.089 (3)	125
N1—H1⋯O1^i^	0.86	2.02	2.825 (2)	156
N3—H3*B*⋯O2^ii^	0.90	2.34	3.057 (3)	137
N4—H4*A*⋯O1^ii^	0.90	2.27	2.979 (3)	136
C6—H6⋯O2^iii^	0.93	2.39	3.293 (3)	165

Symmetry codes: (i) 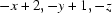; (ii) 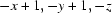; (iii) 
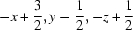
.

**Fig. 1.**
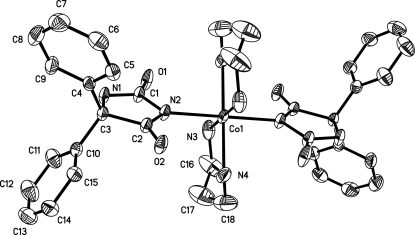
The molecular structure of the title complex showing displacement ellipsoids drawn at the 30% probability level. H-atom are omitted for clarity. Unlabelled atoms are related to the labelled atoms by the symmetry operation 1-x, 1-y, -z.

## supplementary crystallographic information

### Comment

5,5-Diphenylimidazoline-2,4-dione (phenytoin) is a widely used drug in the treatment of epilepsy and an excellent ligand for transition metal complexes (Milne *et al.*, 1999; Akitsu *et al.*, 1997; Akitsu & Einaga, 2005). As a continuation of our research devoted to the design and synthesis of complexes with 5,5-diphenylhydantoinate (Hu *et al.*, 2006, 2007, 2009), we report here the crystal structure of the title compound.

The title compound (Fig. 1) consists of [Co(pht)_2_(pa)_2_] (Hpht = 5,5-diphenylhydantoin; pa = 1,3-propylendiamine) neutral complex molecules having crystallographically imposed inversion symmetry. The Co atom is coordinated by two nitrogen atoms from two pht anions and four nitrogen atoms from two pa ligands in a distorted octahedral CoN_6_ coordination geometry. The Co—N bond distances lie in the range 2.153 (2)–2.180 (2) Å. The imidazole ring (N1/N2/C1–C3) is approximately planar (maximum deviation 0.025 (3) Å for atoms C3) and forms dihedral angles of 62.26 (8) and 57.47 (9)° with the C4–C9 and C10–C15 phenyl rings, respectively. In the crystal structure, intra- and intermolecular N—H···O and C—H···O hydrogen bonds are observed (Table 1), forming a three-dimensional network.

### Experimental

To a solution of 5,5-diphenylhydantoin (1.00 mmol) in methanol (10 ml) was added cobalt(II) acetate tetrahydrate (0.5 mmol) and a solution of 1,3-propylendiamine (1 mmol) in methanol (10 ml). Then the mixture was sealed in a 25 ml stainless steel vessel with Teflon liner and heated to 393 K for 50 h, the fill rate being 80%. After cooling to room temperature, orange single crystals of the title compound suitable for X-ray analysis were obtained.

### Refinement

All H atoms were placed at calculated positions, with N—H = 0.86–0.90 Å, C—H = 0.93–0.97Å, and refined as riding, with *U*_iso_(H) = 1.2 *U*_eq_(C, N).

### Crystal data

**Table:**

[Co(C_15_H_11_N_2_O_2_)_2_(C_3_H_10_N_2_)_2_]	*F*(000) = 746
*M**_r_* = 709.71	*D*_x_ = 1.323 Mg m^−^^3^
Monoclinic, *P*2_1_/*n*	Melting point = 534–536 K
Hall symbol: -P 2yn	Mo *K*α radiation, λ = 0.71073 Å
*a* = 10.0368 (12) Å	Cell parameters from 4059 reflections
*b* = 8.7865 (9) Å	θ = 2.5–27.9°
*c* = 20.684 (2) Å	µ = 0.53 mm^−^^1^
β = 102.363 (2)°	*T* = 298 K
*V* = 1781.8 (3) Å^3^	Block, orange
*Z* = 2	0.50 × 0.46 × 0.45 mm

### Data collection

**Table:**

Siemens SMART CCD area-detector diffractometer	3130 independent reflections
Radiation source: fine-focus sealed tube	2536 reflections with *I* > 2σ(*I*)
graphite	*R*_int_ = 0.026
phi and ω scans	θ_max_ = 25.0°, θ_min_ = 2.0°
Absorption correction: multi-scan (*SADABS*; Sheldrick, 1996)	*h* = −11→11
*T*_min_ = 0.777, *T*_max_ = 0.796	*k* = −10→10
8632 measured reflections	*l* = −19→24

### Refinement

**Table:**

Refinement on *F*^2^	Secondary atom site location: difference Fourier map
Least-squares matrix: full	Hydrogen site location: inferred from neighbouring sites
*R*[*F*^2^ > 2σ(*F*^2^)] = 0.036	H-atom parameters constrained
*wR*(*F*^2^) = 0.097	*w* = 1/[σ^2^(*F*_o_^2^) + (0.0364*P*)^2^ + 1.1953*P*] where *P* = (*F*_o_^2^ + 2*F*_c_^2^)/3
*S* = 1.05	(Δ/σ)_max_ < 0.001
3130 reflections	Δρ_max_ = 0.24 e Å^−^^3^
224 parameters	Δρ_min_ = −0.22 e Å^−^^3^
0 restraints	Extinction correction: *SHELXL97* (Sheldrick, 2008), Fc^*^=kFc[1+0.001xFc^2^λ^3^/sin(2θ)]^-1/4^
Primary atom site location: structure-invariant direct methods	Extinction coefficient: 0.0318 (15)

### Special details

**Table:**

Geometry. All e.s.d.'s (except the e.s.d. in the dihedral angle between two l.s. planes) are estimated using the full covariance matrix. The cell e.s.d.'s are taken into account individually in the estimation of e.s.d.'s in distances, angles and torsion angles; correlations between e.s.d.'s in cell parameters are only used when they are defined by crystal symmetry. An approximate (isotropic) treatment of cell e.s.d.'s is used for estimating e.s.d.'s involving l.s. planes.
Refinement. Refinement of *F*^2^ against ALL reflections. The weighted *R*-factor *wR* and goodness of fit *S* are based on *F*^2^, conventional *R*-factors *R* are based on *F*, with *F* set to zero for negative *F*^2^. The threshold expression of *F*^2^ > σ(*F*^2^) is used only for calculating *R*-factors(gt) *etc*. and is not relevant to the choice of reflections for refinement. *R*-factors based on *F*^2^ are statistically about twice as large as those based on *F*, and *R*- factors based on ALL data will be even larger.

### Fractional atomic coordinates and isotropic or equivalent isotropic displacement parameters (Å^2^)

**Table:**

	*x*	*y*	*z*	*U*_iso_*/*U*_eq_	
Co1	0.5000	0.5000	0.0000	0.03637 (17)	
N1	0.92944 (16)	0.6118 (2)	0.05822 (9)	0.0418 (5)	
H1	1.0091	0.6117	0.0491	0.050*	
N2	0.70243 (16)	0.5782 (2)	0.04395 (9)	0.0412 (5)	
N3	0.5249 (2)	0.5748 (3)	−0.09720 (10)	0.0615 (6)	
H3A	0.6148	0.5873	−0.0949	0.074*	
H3B	0.4972	0.4981	−0.1258	0.074*	
N4	0.4168 (2)	0.7172 (3)	0.02120 (11)	0.0583 (6)	
H4A	0.3484	0.6978	0.0419	0.070*	
H4B	0.4822	0.7657	0.0505	0.070*	
O1	0.81717 (15)	0.4817 (2)	−0.03302 (8)	0.0642 (6)	
O2	0.66668 (15)	0.6952 (2)	0.13808 (8)	0.0539 (5)	
C1	0.8178 (2)	0.5516 (3)	0.01896 (11)	0.0435 (6)	
C2	0.7420 (2)	0.6519 (3)	0.10189 (11)	0.0392 (5)	
C3	0.89906 (19)	0.6768 (3)	0.11802 (10)	0.0350 (5)	
C4	0.9728 (2)	0.5888 (3)	0.17903 (11)	0.0370 (5)	
C5	0.9063 (3)	0.5142 (3)	0.22210 (12)	0.0478 (6)	
H5	0.8117	0.5177	0.2147	0.057*	
C6	0.9794 (3)	0.4346 (3)	0.27608 (14)	0.0630 (7)	
H6	0.9333	0.3851	0.3045	0.076*	
C7	1.1180 (4)	0.4283 (4)	0.28789 (15)	0.0725 (9)	
H7	1.1664	0.3750	0.3242	0.087*	
C8	1.1861 (3)	0.5014 (4)	0.24573 (17)	0.0729 (9)	
H8	1.2808	0.4970	0.2534	0.087*	
C9	1.1138 (2)	0.5813 (3)	0.19190 (13)	0.0555 (7)	
H9	1.1607	0.6309	0.1639	0.067*	
C10	0.9329 (2)	0.8457 (3)	0.12697 (11)	0.0405 (5)	
C11	1.0033 (3)	0.9222 (3)	0.08683 (14)	0.0627 (7)	
H11	1.0304	0.8712	0.0524	0.075*	
C12	1.0341 (4)	1.0768 (4)	0.09782 (17)	0.0823 (10)	
H12	1.0823	1.1282	0.0708	0.099*	
C13	0.9935 (3)	1.1521 (4)	0.14804 (17)	0.0768 (10)	
H13	1.0125	1.2553	0.1545	0.092*	
C14	0.9256 (3)	1.0773 (3)	0.18856 (16)	0.0663 (8)	
H14	0.8990	1.1289	0.2230	0.080*	
C15	0.8962 (2)	0.9249 (3)	0.17859 (13)	0.0514 (6)	
H15	0.8508	0.8741	0.2069	0.062*	
C16	0.4548 (6)	0.7161 (5)	−0.12703 (18)	0.1266 (19)	
H16A	0.3618	0.6906	−0.1486	0.152*	
H16B	0.5005	0.7526	−0.1609	0.152*	
C17	0.4511 (6)	0.8423 (5)	−0.0788 (2)	0.1254 (17)	
H17A	0.5435	0.8589	−0.0540	0.150*	
H17B	0.4228	0.9343	−0.1039	0.150*	
C18	0.3650 (5)	0.8236 (4)	−0.0320 (2)	0.1069 (15)	
H18A	0.2760	0.7890	−0.0555	0.128*	
H18B	0.3529	0.9220	−0.0128	0.128*	

### Atomic displacement parameters (Å^2^)

**Table:**

	*U*^11^	*U*^22^	*U*^33^	*U*^12^	*U*^13^	*U*^23^
Co1	0.0217 (2)	0.0489 (3)	0.0374 (3)	0.00008 (18)	0.00402 (16)	−0.0121 (2)
N1	0.0222 (9)	0.0644 (13)	0.0398 (10)	−0.0066 (8)	0.0089 (7)	−0.0201 (9)
N2	0.0228 (9)	0.0589 (13)	0.0419 (10)	−0.0021 (8)	0.0073 (7)	−0.0200 (9)
N3	0.0480 (12)	0.0901 (18)	0.0444 (12)	−0.0201 (12)	0.0055 (9)	−0.0083 (12)
N4	0.0365 (11)	0.0634 (15)	0.0690 (15)	0.0073 (10)	−0.0021 (10)	−0.0241 (12)
O1	0.0278 (8)	0.1144 (17)	0.0514 (10)	−0.0071 (9)	0.0108 (7)	−0.0475 (11)
O2	0.0294 (8)	0.0796 (13)	0.0547 (10)	−0.0015 (8)	0.0131 (7)	−0.0340 (9)
C1	0.0249 (10)	0.0628 (16)	0.0426 (13)	−0.0021 (10)	0.0068 (9)	−0.0168 (11)
C2	0.0266 (10)	0.0497 (14)	0.0410 (12)	−0.0001 (10)	0.0068 (9)	−0.0134 (11)
C3	0.0243 (10)	0.0445 (13)	0.0361 (11)	−0.0024 (9)	0.0062 (8)	−0.0114 (10)
C4	0.0320 (11)	0.0368 (13)	0.0410 (12)	−0.0042 (9)	0.0056 (9)	−0.0123 (10)
C5	0.0460 (13)	0.0482 (15)	0.0509 (14)	−0.0082 (11)	0.0143 (11)	−0.0066 (12)
C6	0.084 (2)	0.0521 (17)	0.0553 (17)	−0.0086 (15)	0.0196 (15)	0.0014 (14)
C7	0.086 (2)	0.062 (2)	0.0620 (19)	0.0063 (17)	−0.0028 (16)	0.0124 (16)
C8	0.0444 (15)	0.083 (2)	0.081 (2)	0.0034 (15)	−0.0087 (14)	0.0110 (18)
C9	0.0341 (12)	0.0646 (18)	0.0640 (17)	−0.0067 (12)	0.0022 (11)	0.0078 (14)
C10	0.0319 (11)	0.0443 (14)	0.0405 (12)	−0.0013 (10)	−0.0032 (9)	−0.0016 (10)
C11	0.0733 (18)	0.0644 (19)	0.0480 (15)	−0.0186 (15)	0.0075 (13)	−0.0012 (14)
C12	0.097 (3)	0.071 (2)	0.071 (2)	−0.029 (2)	0.0016 (18)	0.0202 (18)
C13	0.089 (2)	0.0456 (17)	0.081 (2)	−0.0035 (16)	−0.0157 (18)	−0.0020 (17)
C14	0.0643 (18)	0.0494 (18)	0.079 (2)	0.0019 (14)	0.0018 (15)	−0.0148 (16)
C15	0.0469 (14)	0.0455 (15)	0.0602 (16)	−0.0001 (12)	0.0077 (12)	−0.0128 (13)
C16	0.230 (6)	0.074 (3)	0.057 (2)	−0.036 (3)	−0.013 (3)	0.012 (2)
C17	0.201 (6)	0.072 (3)	0.096 (3)	−0.007 (3)	0.015 (3)	0.013 (3)
C18	0.120 (3)	0.070 (2)	0.105 (3)	0.030 (2)	−0.032 (3)	−0.018 (2)

### Geometric parameters (Å, °)

**Table:**

Co1—N2^i^	2.1531 (16)	C6—C7	1.361 (4)
Co1—N2	2.1531 (16)	C6—H6	0.9300
Co1—N4^i^	2.165 (2)	C7—C8	1.377 (4)
Co1—N4	2.165 (2)	C7—H7	0.9300
Co1—N3^i^	2.180 (2)	C8—C9	1.384 (4)
Co1—N3	2.180 (2)	C8—H8	0.9300
N1—C1	1.343 (3)	C9—H9	0.9300
N1—C3	1.453 (3)	C10—C11	1.375 (3)
N1—H1	0.8600	C10—C15	1.389 (3)
N2—C2	1.345 (3)	C11—C12	1.401 (5)
N2—C1	1.385 (3)	C11—H11	0.9300
N3—C16	1.494 (5)	C12—C13	1.366 (5)
N3—H3A	0.9000	C12—H12	0.9300
N3—H3B	0.9000	C13—C14	1.358 (4)
N4—C18	1.452 (4)	C13—H13	0.9300
N4—H4A	0.9000	C14—C15	1.378 (4)
N4—H4B	0.9000	C14—H14	0.9300
O1—C1	1.237 (3)	C15—H15	0.9300
O2—C2	1.232 (2)	C16—C17	1.498 (6)
C2—C3	1.555 (3)	C16—H16A	0.9700
C3—C10	1.525 (3)	C16—H16B	0.9700
C3—C4	1.529 (3)	C17—C18	1.438 (6)
C4—C9	1.384 (3)	C17—H17A	0.9700
C4—C5	1.387 (3)	C17—H17B	0.9700
C5—C6	1.387 (4)	C18—H18A	0.9700
C5—H5	0.9300	C18—H18B	0.9700
			
N2^i^—Co1—N2	180.00 (10)	C4—C5—H5	119.6
N2^i^—Co1—N4^i^	90.18 (7)	C7—C6—C5	120.6 (3)
N2—Co1—N4^i^	89.82 (7)	C7—C6—H6	119.7
N2^i^—Co1—N4	89.82 (7)	C5—C6—H6	119.7
N2—Co1—N4	90.18 (7)	C6—C7—C8	119.6 (3)
N4^i^—Co1—N4	180.00 (12)	C6—C7—H7	120.2
N2^i^—Co1—N3^i^	90.51 (7)	C8—C7—H7	120.2
N2—Co1—N3^i^	89.49 (7)	C7—C8—C9	120.0 (3)
N4^i^—Co1—N3^i^	92.65 (10)	C7—C8—H8	120.0
N4—Co1—N3^i^	87.35 (10)	C9—C8—H8	120.0
N2^i^—Co1—N3	89.49 (7)	C4—C9—C8	121.2 (3)
N2—Co1—N3	90.51 (7)	C4—C9—H9	119.4
N4^i^—Co1—N3	87.35 (10)	C8—C9—H9	119.4
N4—Co1—N3	92.65 (10)	C11—C10—C15	118.3 (2)
N3^i^—Co1—N3	180.00 (13)	C11—C10—C3	122.2 (2)
C1—N1—C3	111.68 (16)	C15—C10—C3	119.5 (2)
C1—N1—H1	124.2	C10—C11—C12	120.0 (3)
C3—N1—H1	124.2	C10—C11—H11	120.0
C2—N2—C1	107.61 (17)	C12—C11—H11	120.0
C2—N2—Co1	126.99 (13)	C13—C12—C11	120.2 (3)
C1—N2—Co1	125.27 (14)	C13—C12—H12	119.9
C16—N3—Co1	119.6 (2)	C11—C12—H12	119.9
C16—N3—H3A	107.4	C14—C13—C12	120.4 (3)
Co1—N3—H3A	107.4	C14—C13—H13	119.8
C16—N3—H3B	107.4	C12—C13—H13	119.8
Co1—N3—H3B	107.4	C13—C14—C15	119.9 (3)
H3A—N3—H3B	106.9	C13—C14—H14	120.1
C18—N4—Co1	120.3 (2)	C15—C14—H14	120.1
C18—N4—H4A	107.2	C14—C15—C10	121.2 (3)
Co1—N4—H4A	107.2	C14—C15—H15	119.4
C18—N4—H4B	107.2	C10—C15—H15	119.4
Co1—N4—H4B	107.2	N3—C16—C17	114.6 (3)
H4A—N4—H4B	106.9	N3—C16—H16A	108.6
O1—C1—N1	124.70 (19)	C17—C16—H16A	108.6
O1—C1—N2	123.96 (19)	N3—C16—H16B	108.6
N1—C1—N2	111.35 (19)	C17—C16—H16B	108.6
O2—C2—N2	125.96 (19)	H16A—C16—H16B	107.6
O2—C2—C3	123.48 (19)	C18—C17—C16	117.9 (4)
N2—C2—C3	110.56 (17)	C18—C17—H17A	107.8
N1—C3—C10	113.85 (19)	C16—C17—H17A	107.8
N1—C3—C4	110.53 (17)	C18—C17—H17B	107.8
C10—C3—C4	109.62 (17)	C16—C17—H17B	107.8
N1—C3—C2	98.61 (15)	H17A—C17—H17B	107.2
C10—C3—C2	110.65 (17)	C17—C18—N4	114.5 (4)
C4—C3—C2	113.27 (18)	C17—C18—H18A	108.6
C9—C4—C5	117.8 (2)	N4—C18—H18A	108.6
C9—C4—C3	118.5 (2)	C17—C18—H18B	108.6
C5—C4—C3	123.67 (19)	N4—C18—H18B	108.6
C6—C5—C4	120.7 (2)	H18A—C18—H18B	107.6
C6—C5—H5	119.6		
			
N4^i^—Co1—N2—C2	−133.8 (2)	N2—C2—C3—C4	−114.1 (2)
N4—Co1—N2—C2	46.2 (2)	N1—C3—C4—C9	59.8 (3)
N3^i^—Co1—N2—C2	−41.2 (2)	C10—C3—C4—C9	−66.5 (3)
N3—Co1—N2—C2	138.8 (2)	C2—C3—C4—C9	169.3 (2)
N4^i^—Co1—N2—C1	41.5 (2)	N1—C3—C4—C5	−119.5 (2)
N4—Co1—N2—C1	−138.5 (2)	C10—C3—C4—C5	114.2 (2)
N3^i^—Co1—N2—C1	134.1 (2)	C2—C3—C4—C5	−9.9 (3)
N3—Co1—N2—C1	−45.9 (2)	C9—C4—C5—C6	−0.2 (4)
N2^i^—Co1—N3—C16	75.1 (2)	C3—C4—C5—C6	179.1 (2)
N2—Co1—N3—C16	−104.9 (2)	C4—C5—C6—C7	0.1 (4)
N4^i^—Co1—N3—C16	165.3 (2)	C5—C6—C7—C8	−0.2 (5)
N4—Co1—N3—C16	−14.7 (2)	C6—C7—C8—C9	0.3 (5)
N2^i^—Co1—N4—C18	−72.1 (3)	C5—C4—C9—C8	0.4 (4)
N2—Co1—N4—C18	107.9 (3)	C3—C4—C9—C8	−179.0 (3)
N3^i^—Co1—N4—C18	−162.7 (3)	C7—C8—C9—C4	−0.5 (5)
N3—Co1—N4—C18	17.3 (3)	N1—C3—C10—C11	−8.7 (3)
C3—N1—C1—O1	−175.7 (3)	C4—C3—C10—C11	115.7 (2)
C3—N1—C1—N2	4.5 (3)	C2—C3—C10—C11	−118.6 (2)
C2—N2—C1—O1	177.8 (3)	N1—C3—C10—C15	173.60 (19)
Co1—N2—C1—O1	1.7 (4)	C4—C3—C10—C15	−62.0 (3)
C2—N2—C1—N1	−2.4 (3)	C2—C3—C10—C15	63.6 (3)
Co1—N2—C1—N1	−178.51 (16)	C15—C10—C11—C12	−1.1 (4)
C1—N2—C2—O2	179.5 (3)	C3—C10—C11—C12	−178.9 (2)
Co1—N2—C2—O2	−4.5 (4)	C10—C11—C12—C13	−0.5 (5)
C1—N2—C2—C3	−0.4 (3)	C11—C12—C13—C14	1.4 (5)
Co1—N2—C2—C3	175.61 (15)	C12—C13—C14—C15	−0.7 (5)
C1—N1—C3—C10	−121.4 (2)	C13—C14—C15—C10	−1.0 (4)
C1—N1—C3—C4	114.7 (2)	C11—C10—C15—C14	1.8 (4)
C1—N1—C3—C2	−4.2 (2)	C3—C10—C15—C14	179.6 (2)
O2—C2—C3—N1	−177.2 (2)	Co1—N3—C16—C17	38.1 (5)
N2—C2—C3—N1	2.7 (2)	N3—C16—C17—C18	−70.4 (6)
O2—C2—C3—C10	−57.6 (3)	C16—C17—C18—N4	74.0 (5)
N2—C2—C3—C10	122.3 (2)	Co1—N4—C18—C17	−44.7 (4)
O2—C2—C3—C4	66.0 (3)		

Symmetry codes: (i) −*x*+1, −*y*+1, −*z*.

### Hydrogen-bond geometry (Å, °)

**Table:**

*D*—H···*A*	*D*—H	H···*A*	*D*···*A*	*D*—H···*A*
N3—H3A···O1	0.90	2.35	3.061 (3)	136.
N4—H4B···O2	0.90	2.38	3.095 (3)	137.
C5—H5···O2	0.93	2.46	3.089 (3)	125
N1—H1···O1^ii^	0.86	2.02	2.825 (2)	156.
N3—H3B···O2^i^	0.90	2.34	3.057 (3)	137.
N4—H4A···O1^i^	0.90	2.27	2.979 (3)	136.
C6—H6···O2^iii^	0.93	2.39	3.293 (3)	165

Symmetry codes: (ii) −*x*+2, −*y*+1, −*z*; (i) −*x*+1, −*y*+1, −*z*; (iii) −*x*+3/2, *y*−1/2, −*z*+1/2.
